# Impact of Fruit Zone Leaf Removal on Anthocyanin Stability in Wine During Bottle Ageing

**DOI:** 10.17113/ftb.63.03.25.8804

**Published:** 2025-09-24

**Authors:** Marina Pavlović, Zoran Zorić, Šime Marcelić, Maja Repajić, Iva Šikuten, Darko Preiner

**Affiliations:** 1 University of Zadar, Department of Ecology, Agronomy and Aquaculture, Trg kneza Višeslava 9, 23000 Zadar, Croatia; 2 University of Zagreb Faculty of Food Technology and Biotechnology, Pierottijeva 6, 10000 Zagreb, Croatia; 3 University of Zagreb Faculty of Agriculture, Svetošimunska cesta 25, 10000 Zagreb, Croatia; 4 The Centre of Excellence for Biodiversity and Molecular Plant Breeding, Svetošimunska 25, 10000 Zagreb, Croatia

**Keywords:** red wine, anthocyanin stability, phenolic compounds, wine ageing, Mediterranean climate

## Abstract

**Research background:**

Anthocyanins, the most abundant pigments in red wine, play an important role in the visual aspect of wine sensory properties. However, due to their unstable nature, their ability to polymerise with tannins is important for colour stability. Their content varies with grapevine variety, growing conditions, viticultural and winemaking practices. Leaf removal, a common viticultural practice, enhances anthocyanin accumulation in red grapevines, and partial fruit zone leaf removal at different phenological stages can significantly influence the anthocyanin content of grapes and wine. This two-year study examined how two different times of fruit zone leaf removal at different phenological stages affect the initial anthocyanin content in wine and their stability during ageing in Merlot, Syrah and Cabernet Sauvignon wines grown in a Mediterranean climate.

**Experimental approach:**

Partial leaf removal was applied during flowering and during vérasion and compared with untreated control. The wines obtained from all treatments and varieties were bottled two months after the end of fermentation, and then stored and matured under the cellar conditions for one year. To determine the influence of different times of leaf removal on the concentration of anthocyanins and their stability in the wine, the wines were analysed immediately after bottling and again after 6 and 12 months of storage. For the determination of all phenolic compounds, high-performance liquid chromatography (HPLC) was used.

**Results and conclusions:**

Leaf removal treatments increased the concentration of anthocyanins in all three cultivars. The obtained results showed that malvidin-3-*O*-glucoside (Mal-3-Glc) was the most abundant individual anthocyanin, while the most unstable anthocyanin was petunidin-3-*O*-coumaroyl glucoside (Pet-3-Coum-Glc). Initial concentration of total anthocyanins in all wine samples was significantly affected by different conditions in the two years of study, but with a significant effect of the defoliation treatments. Anthocyanin concentration decreased during the ageing of the wine, and the degradation of anthocyanins ranged from 36 to 90 %. The stability of anthocyanins in wine was most influenced by ageing time, while year and treatment had no influence. The concentration of total phenolic acids increased during wine ageing, while the concentration of total flavonol glycosides (TFG) decreased in all wine samples except Merlot from 2016.

**Novelty and scientific contribution:**

The results of this study contribute to a better understanding of the stability of increased concentrations of anthocyanins in wines during ageing obtained by the practice of grapevine leaf removal in the vineyard.

## INTRODUCTION

Anthocyanins are water-soluble pigments that occur in the vacuoles of the skin cells responsible for the red colour of the grape skin and are responsible for the intense colour of red wines (*1*). Besides being colour pigments, anthocyanins have other roles, such as protecting plants from excessive sun and UV radiation, scavenging free radicals, increasing antioxidant capacity and protecting against numerous pathogenic organisms (*2*).

Anthocyanin biosynthesis is one of the most important biochemical processes during the growth and development of red grapevine cultivars. The accumulation of anthocyanins in the berry skin is influenced by agroecological factors, the most important of which are grapevine variety, climate, soil conditions, canopy management irrigation and yield (*3*).

The accumulation of anthocyanins in grapes begins at vérasion and is characterized by a rapid increase in concentration in the first stage, followed by a slower accumulation or even a drop in the concentration by the end of ripening (*4*, *5*).

Leaf removal in the cluster zone, as a common viticultural practice, has a significant role in the synthesis of polyphenols in grapes. Due to excessive insolation and UV radiation, the plant synthesises anthocyanins as a defence mechanism (*2*). Light positively affects the accumulation of anthocyanins in the berry (*6*, *7*). Excessive lighting can, indirectly by heating the berries, lead to their reduction (*8*, *9*) because temperatures above 30 °C cause inhibition of anthocyanin synthesis (*10*). This phenomenon significantly depends on the variety, so in certain varieties, partial defoliation positively affects the synthesis of polyphenols (*11*, *12*) without the negative influence of increased temperature (*8*, *13*).

Regarding the time of leaf removal, the impact on specific grape qualitative (sugar concentration, titratable acidity, phenolic compounds, *etc*.) and quantitative parameters (yield), and thus on the wine, is different. The early leaf removal, before or during flowering, has the effect of increasing the concentration of total anthocyanins, as shown in different grapevine varieties such as Tempranillo (*12*), Carignan (*14*), Barbera and Lambrusco (*15*). According to Di Profio *et al.* (*16*) partial leaf removal of basal leaves on Merlot, Cabernet Sauvignon and Cabernet franc increases the concentration of total anthocyanins and colour intensity of all three cultivars. By removing the leaves after vérasion, Palliotti *et al.* (*17*) determined that the anthocyanin content was not significantly different from that of the control vines without leaf removal. Late leaf removal, during vérasion, reduces anthocyanin content and increases the negative impact of sunburn, while leaf removal before flowering increases sugar and anthocyanin content (*11*).

The main drawback of anthocyanins is their extremely low stability, which is easily influenced by external factors, such as light and temperature (*18*). Thus, it is extremely important for the red wine colour stability that anthocyanins are found in more stable (glycoside) forms. Anthocyanins are initially found in grapes in monomeric forms. As they are highly reactive in nature, their forms change in various reactions and interactions during winemaking and wine ageing (*1*). The stability of anthocyanins can be achieved in several ways, by copigmentation or polymerisation with flavan-3-ols and procyanidins, creating new pigments and polymeric anthocyanins that significantly affect the stability of wine colour (*19*-*21*). The stability of anthocyanins can be achieved by sugar acylation (*2*) because the rest of the sugar can be acylated with aromatic or aliphatic acids at the C-6 position. Although the initial concentration of anthocyanins in young wines is high immediately after fermentation, due to their instability, the concentration of these acylated anthocyanins drops just after fermentation, and they disappear after a few months (*22*). The concentration of anthocyanins in young wines after fermentation can vary from 100 to 1500 mg/L, depending on the cultivar (*22*).

The aim of this study is to investigate the influence of different times of fruit zone leaf removal on the content and stability of anthocyanins in wines of Merlot, Syrah and Cabernet Sauvignon during ageing in bottles.

## MATERIALS AND METHODS

### Vineyard site, plant material and weather conditions

The research was conducted in 2015 and 2016 on cultivars Merlot, Syrah, and Cabernet Sauvignon. The vineyard is located 20 km north of Zadar (Baštica, Suhovare) in Dalmatia region, subregion Dalmatian hinterland (latitude 44°06´N; longitude 15°13´E) and is a part of the University of Zadar, Croatia. All three grapevine cultivars were grafted on Kober 5BB (*Vitis berlandieri* Planch. × *Vitis riparia* Michx.) rootstock, which was planted in 2007 on anthropogenic soil called regosol with a sandy clay texture. The vines were planted with a spacing of 90 cm within the row and 280 cm between rows (planting density of 4100 vines per ha). All three grapevine cultivars were trained to a vertical shoot position with single-cane-pruned Guyot, leaving about 12 to 14 buds per vine. The basal wire was placed at 100 cm above the ground, with two sets of catch wires positioned 50 and 90 cm above the cordon. The maximum canopy height was 200 cm. The experimental field had no irrigation system and the space between the rows was grassed. The same vineyard management practices were used for all treatments.

The beginning of the main grapevine phenophases was determined visually. Full flowering was estimated when 50 % of the flower caps had fallen off, corresponding to stage 23 according to the modified Eichorn and Lorenz (E-L) system (*23*), while vérasion was estimated when the berries started to brighten in colour, corresponding to stage 35 according to the same scale.

The harvest date was determined by measuring the total soluble solids (Brix), total acids (g/L), and pH. Harvesting began when the total soluble solids were above 19 °Brix. Grapes were harvested manually at different times depending on the grapevine variety and measured parameters. Grapes for each treatment were harvested separately.

Weather conditions, including average temperature and precipitation for both seasons from April to September, were measured by the Croatian Meteorological and Hydrological Service (Weather station Benkovac), 25 km from the experimental vineyard, and the data are shown in Table S1. The weather conditions were reflected at the beginning of flowering and vérasion, and also during the leaf removal treatments. The harvest time differed only by a few days in both years. Merlot was harvested three days earlier in 2016 (22 September) than in 2015 (25 September), probably due to the previously mentioned dry period in July, which affected the slightly earlier harvest. Syrah was harvested on the same day as Merlot in 2015 (25 September), but 8 days later than Merlot in 2016, on 30 September. Cabernet Sauvignon was harvested on 9 October in 2015, and on 13 October in 2016.

### Experimental design

The experiment was a completely randomised block design with three treatments in three replications for each cultivar. Each replication consisted of 15 continuous plants, so there were 135 plants per cultivar and 405 plants in total. The cultivars were in the same vineyard, but one was next to the other, so the experiment was set up in the same way for different grapevine varieties. All treatments were repeated for two years in the same part of the vineyard.

The three treatments were: (*i*) leaf removal during flowering (full flowering, 50 % open flowers); (*ii*) leaf removal during vérasion (beginning of vérasion, 30 % of the berries are coloured), and (*iii*) control (C) - without leaf removal. In both leaf removal treatments, the basal leaves were removed up to the height of the last cluster on the shoot (4 to 6 leaves).

### Vinification

Manually harvested grapes were destemmed and crushed separately for each variety and treatment and placed in an open plastic container (100 L) for maceration and fermentation. All vinifications were sulphited with 5 g K_2_S_2_O_5_ per 100 L and after a few hours, *Saccharomyces cerevisiae* yeast (ICV D254; Lallemand, Montreal, Canada) was inoculated at a concentration of 25 g/100 L. The pomace was stirred manually twice a day and the temperature was between 25 and 28 °C. After seven days of maceration and fermentation, the wine was racked and fermentation continued in glass containers. At the end of fermentation, the wine was additionally sulphited with 5 g K_2_S_2_O_5_ per 100 L, racked again and bottled in 0.75-litre bottles two months after the end of fermentation.

Must samples were collected immediately after primary processing, for analysis of total soluble solids, titratable acidity and pH. Total soluble solids in the must were measured using a handheld refractometer (RHB 32 ATC; PR China) (expressed in °Brix) and pH was determined with a pH meter (Lab 860; Schott Instruments; Mainz, Germany). The titratable acidity (g/L) was determined using the colouration pattern volumetric method according to the O.I.V. (*24*).

The wine was stored and matured under the cellar conditions for one year after bottling. Samples for analysis were taken at random in triplicate after bottling, *i.e.* after 0 months and after 6 and 12 months.

### Analysis of phenolic content using HPLC-DAD

The concentration of anthocyanins and other phenolic compounds (phenolic acids, procyanidins, flavan-3-ols and flavonol glycosides) was determined in all wine samples using high-performance liquid chromatography (HPLC). The wine samples were filtered through 0.45-µm syringe filters (Macherey-Nagel GmbH & Co. KG, Duren, Germany) into glass vials and analysed using the HPLC Agilent Inﬁnity 1260 system equipped with an Agilent 1260 photodiode array detector (PDA; Agilent, Santa Clara, CA, USA), an automatic injector and Chemstation software (v. C.01.03) for data processing and instrument control. Phenolic compounds were separated using Luna 100-5C18 column, 5 µm (250 mm×4.6 mm; Phenomenex, Aschaffenburg, Germany). The injection volume was 5 µL, and the solvent composition and gradient conditions were as previously described by Zorić *et al*. (*25*).

All anthocyanins were identiﬁed at *λ*=520 nm by comparing their retention times and absorption spectra with those of authentic standards. All identified compounds were quantified according to the calibration curves of standards. Standards of delphinidin-3-glucoside (Del-3-Glc), cyanidin 3-glucoside (Cy-3-Glc), petunidin-3-glucoside (Pet-3-Glc), peonidin-3-glucoside (Peo-3-Glc) and malvidin-3-glucoside (Mal-3-Glc) were prepared as stock solutions at a concentration of 100 mg/L in methanol acidiﬁed with *φ*(formic acid)=1 %. The stock solutions were diluted to obtain ﬁve concentrations between 20 and 100 mg/L.

Phenolic acids, procyanidins, flavan-3-ols and flavonol glycosides were identified by comparing the retention times and spectral data with those of authentic standards prepared in methanol, namely: chlorogenic acid, caffeic acid, *p*-coumaric acid, gallic acid, procyanidins B1 and B6, epigallocatechin gallate, catechin, quercetin-3-glucoside and kaempferol-3-rutinoside.

All results were expressed as mg/L in the form of mean value±standard deviation.

### Statistical analysis

Statistica v. 14.0 software (*26*) was used for the statistical analysis. Descriptive statistics was used to assess the basic information about the experimental data set, and the data are presented as mean value±S.E. The normality and homoscedasticity of the data were analysed using the Shapiro-Wilk test and Levene’s test, respectively, and were evaluated accordingly by ANOVA coupled with the *post hoc* Tukey’s HSD test with multiple comparisons of mean ranks. A statistically signiﬁcant difference at p≤0.05 was assigned for all tests.

## RESULTS AND DISCUSSION

### Basic chemical parameters of the must

The basic chemical parameters measured in the must of three grape varieties (total soluble solids, titratable acidity and pH) were mainly influenced by the experimental year, while there was no significant difference among varieties and leaf removal treatments (Table 1).

**Table 1:** Soluble solids, titratable acidity and pH influenced by year, cultivar and leaf removal effect

Type of influence	Soluble solid/°Brix	Titratable acidity/(g/L)	pH
Year	p<0.001*	p<0.001*	p<0.001*
2015	(20.3±0.2)^b^	(5.0±0.1)^b^	(3.58±0.02)^a^
2016	(21.4±0.2)^a^	(5.78±0.08)^a^	(3.38±0.02)^b^
Cultivar	p<0.001*	p=0.288	p=0.476
Merlot	(20.4±0.2)^b^	(5.2±0.2)^a^	(3.47±0.03)^a^
Syrah	(20.7±0.2)^b^	(5.4±0.1)^a^	(3.47±0.04)^a^
Cabernet Sauvignon	(21.5±0.4)^a^	(5.56±0.05)^a^	(3.51±0.02)^a^
Leaf removal effect	p=0.387	p=0.942	p=0.621
Control	(20.6±0.3)^a^	(5.4±0.2)^a^	(3.46±0.04)^a^
LRF	(21.1±0.3)^a^	(5.3±0.2)^a^	(3.49±0.03)^a^
LRV	(20.9±0.3)^a^	(5.4±0.2)^a^	(3.50±0.02)^a^

LRF=leaf removal during flowering, LRV=leaf removal during véraison. *Statistically signiﬁcant variable at p≤0.05. Results are expressed as mean value±S.E. Values with different letters in superscript within a column are statistically different at p≤0.05

The two experimental years differed in the average temperature and precipitation during the vegetation period, with 2015 being 0.7 °C warmer and having about 125 mm less precipitation. Furthermore, the ripening period was on average 1.3 °C warmer in August 2015 than in 2016. Higher temperatures have an effect on increased cell respiration, which leads to malic acid breakdown (*27*) and a lower acidity. This was observed in the 2015 samples, which had lower acidity and, consequently, higher pH than the 2016 samples. A similar observation that experimental year has a significant effect on basic chemical parameters, compared to leaf removal treatments, was made by Mosetti *et al.* (*28*) in Sauvignon blanc and Anić *et al.* (*29*) in Merlot, although in some cases, a mild influence of leaf removal treatments on basic chemical parameters was observed (*30*, *31*). The time of leaf removal also had no influence on the basic chemical parameters of the must samples. There is no difference between the treatments in titratable acidity and pH, which is consistent with other research (*32*, *33*).

Defoliation treatments did not affect the increase in total soluble solids, regardless of when they were applied. These observations are consistent with other studies (*29*, *32*, *33*).

### Effect of leaf removal on anthocyanin content in wine

Leaf removal treatments positively influenced the accumulation of anthocyanin in all three grapevine varieties, which was expected and is consistent with other research on different varieties (*29*, *34*, *35*). Similar results were obtained by other authors. For example, in the research on the Italian cultivar Nebbiolo, the concentration of individual anthocyanins and polyphenols depended on the year and climatic conditions. Nevertheless, the total concentration was consistently higher in defoliated samples than in the control (*35*). The effect of the leaf removal on the composition of individual anthocyanins in Merlot, Syrah and Cabernet Sauvignon wine is shown in Table 2. In all three grapevine varieties, malvidin-3-*O*-glucoside (Mal-3-Glc) was the most abundant anthocyanin, with concentration depended on the year and leaf removal treatment, which is consistent with the studies of Shi *et al*. (*36*). The second most abundant anthocyanin in all three varieties was malvidin-3-*O*-acetyl-glucoside (Mal-3-Ac-Glc).

**Table 2:** The effect of leaf removal on the composition of anthocyanins in Merlot, Syrah and Cabernet Sauvignon wine shown as average values of three ageing periods

Wine	Year		*γ*/(mg/L)						
			Pet-3-Glc	Peo-3-Glc	Malv-3-Glc	Pet-3-Coum-Glc	Peo-3-Coum-Glc	Mal-3-Ac-Glc	Mal-3-Coum-Glc
Merlot	2015	Treatment							
		Control	(1.9±0.5)^c^	(0.8±0.2)^a^	(35.9±5.4)^b^	(0.5±0.1)^a^	(0.97±0.06)^b^	(13.3±2.2)^b^	(6.9±1.3)^c^
		LRF	(2.9±0.9)^a^	(0.8±0.2)^a^	(45.1±11.9)^a^	(0.7±0.3)^a^	(1.5±0.1)^a^	(16.9±4.9)^a^	(9.2±3.0)^a^
		LRV	(2.4±0.7)^b^	(0.8±0.2)^a^	(42.1±8.5)^a,b^	(0.6±0.2)^a^	(1.5±0.1)^a,b^	(16.3±3.6)^a,b^	(8.2±2.1)^b^
		Significance	***	n.s.	**	n.s.	**	**	***
	2016	Control	(2.1±0.2)^c^	(1.3±0.5)^a^	(39.3±5.4)^c^	n.d.	(0.62±0.2)^a^	(12.1±2.4)^c^	(7.0±1.3)^c^
		LRF	(2.9±0.1)^b^	(1.7±0.6)^a^	(52.5±4.1)^b^	(0.4±0.2)^a^	(0.61±0.2)^a^	(15.3±2.2)^b^	(10.1±1.0)^a,b^
		LRV	(4.2±0.2)^a^	(1.9±0.6)^a^	(63.8±4.3)^a^	(0.5±0.3)^a^	(0.8±0.2)^a^	(21.8±2.5)^a^	(11.4±0.9)^a^
		Significance	***	n.s.	***	n.s.	n.s.	***	**
Syrah	2015	Treatment							
		Control	(3.6±0.6)^b^	(3.0±0.4)^b^	(56.6±7.3)^c^	(1.6±0.2)^b^	(2.8±0.5)^b^	(24.5±3.8)^b^	(13.2±2.0)^b^
		LRF	(3.8±0.7)^b^	(2.6±0.4)^c^	(63.6±9.6)^b^	(2.1±0.5)^a^	(2.8±0.4)^b^	(26.7±4.6)^a,b^	(13.8±2.1)^b^
		LRV	(4.3±0.8)^a^	(3.6±0.5)^a^	(73.4±10.5)^a^	(1.8±0.2)^b^	(3.2±0.7)^a^	(31.0±5.4)^a^	(17.7±2.9)^a^
		Signif.	**	***	***	**	**	**	**
	2016	Control	(2.5±0.3)^a,b^	(1.6±0.2)^a,b^	(49.9±4.8)^a,b^	n.d.	(1.7±0.5)^a,b^	(19.5±3.2)^a,b^	(11.3±1.6)^a,b^
		LRF	(2.4±0.4)^b^	(1.1±0.2)^b^	(45.9±6.5)^b^	n.d.	(1.2±0.3)^b^	(17.3±3.2)^b^	(9.3±1.8)^b^
		LRV	(3.2±0.4)^a^	(1.7±0.2)^a^	(54.4±5.5)^a^	n.d.	(2.4±0.5)^a^	(20.8±3.5)^a^	(12.1±1.8)^a^
		Significance	**	**	**	-	**	**	**
Cabernet Sauvignon	2015	Treatment							
		Control	(1.8±0.5)^a^	(0.3±0.1)^a^	(59.5±10.7)^a^	(0.8±0.2)^b^	(0.9±0.2)^a^	(26.6±5.0)^a^	(5.2±1.2)^a^
		LRF	(1.8±0.5)^a^	(0.4±0.6)^a^	(54.4±9.7)^a,b^	(1.16±0.05)^a,b^	(1.0±0.1)^a^	(23.7±4.1)^a,b^	(3.8±0.9)^b^
		LRV	(1.6±0.4)^b^	(0.5±0.4)^a^	(47.5±6.6)^b^	(1.3±0.1)^a^	(0.83±0.08)^a^	(20.07±3.06)^b^	(4.5±0.8)^a,b^
		Significance	**	n.s.	**	**	n.s.	**	**
	2016	Control	(0.4±0.2)^b^	(2.3±0.2)^b^	(59.1±3.4)^a,b^	n.d.	(0.7±0.2)^a^	(27.4±1.8)^a^	(5.9±0.4)^a^
		LRF	(0.7±1.0)^a^	(4.0±0.2)^a^	(67.1±4.0)^a^	n.d.	(1.1±0.3)^a^	(27.7±1.9)^a^	(5.3±0.4)^a^
		LRV	(0.6±0.9)^a^	(2.7±0.4)^b^	(53.1±6.4)^b^	n.d.	(0.6±0.2)^a^	(21.5±3.0)^b^	(5.4±0.8)^a^
		Significance	**	**	**	-	n.s.	**	n.s.

Data were analysed using one-way ANOVA model and presented as mean value±S.D., *N*=9; ns, ** and *** indicate not significant, significant at p<0.01 and p>0.0001, respectively. LRF=leaf removal during flowering, LRV=leaf removal during véraison. Mean values with different letters in superscript are significantly different within treatment. Abbreviations: Pet-3-Glc=petunidin-3-O-glucoside, Peo-3-Glc=peonidin-3-O-glucoside, Malv-3-Glc=malvidin-3-O-glucoside, Pet-3-Coum-Glc=petunidin-3-O-(coumaroyl) glucoside, Peo-3-Coum-Glc=peonidin-3-O-(coumaroyl) glucoside, Mal-3-Ac-Glc=malvidin-3-O-(acetyl) glucoside, Mal-3-Coum-Glc=malvidin-3-O-(coumaroyl) glucoside

The influence of the time of defoliation on the concentration of total and individual anthocyanins depended on the variety and the year (Table S2, Table S3 and Table S4). Similar effects were observed on Merlot, Pinot noir and Gamay, where the experimental year had an important role in the success of the leaf removal treatments (*32*-*37*). In both years, there was a significant influence of defoliation treatment on the concentration of Pet-3-Glc, Malv-3-Glc, Peo-3-Coum-Glc, Mal-3-Ac-Glc and Mal-3-Coum-Glc in Merlot, while defoliation had no significant effect on the remaining individual anthocyanins. In contrast to Merlot, the defoliation treatments consistently increased the anthocyanin content of Syrah in both years, with defoliation during vérasion having the greatest effect. Unlike Merlot, the effect of defoliation did not vary significantly between years. In 2016, Pet-3-Coum-Glc was undetectable in all treatments. In Cabernet Sauvignon, the effect of leaf removal depended on the experimental year. Only defoliation during flowering in 2016 had a significant influence on the individual anthocyanin concentration in Cabernet Sauvignon wines, while the control wines had the highest anthocyanin content in 2015 (Table 2).

Regarding the time of leaf removal, different results have been reported. According to some studies, a higher concentration of anthocyanins was found after early leaf removal during flowering than after leaf removal during vérasion (*11*, *38*), which is similar to our results in Merlot wines from 2015 and Cabernet Sauvignon wines from 2016 (Table 3 and Table 4). In contrast, Merlot from 2016 and Syrah from both years were found to have a significant influence on the increase in the concentration of total anthocyanins at defoliation during vérasion (Table 3 and Table 5). The highest concentration of total anthocyanins in Cabernet Sauvignon from 2015 was found in the control sample (Table 4).

**Table 3:** The effect of leaf removal on the phenolic composition of Merlot wine

Year		*γ*/(mg/L)				
	TA	TPA	TPro	TFL-3-ols	TFG
2015	Treatment					
	Control	64.23^c^	52.40^c^	85.95^b^	19.76^c^	26.15^c^
	LRF	77.13^a^	61.29^b^	80.98^c^	22.31^b^	36.78^a^
	LRV	71.87^b^	62.81^a^	95.45^a^	24.18^a^	33.54^b^
	Significance	***	***	***	***	***
	*t*/month					
	0	121.94^a^	58.75^a^	102.87^a^	25.06^a^	45.08^a^
	6	64.59^b^	57.98^a^	86.27^b^	22.15^b^	31.32^b^
	12	20.29^c^	59.05^a^	71.37^c^	18.46^c^	17.96^c^
	Significance	***	ns	***	***	***
	T×P	***	ns	***	***	***
2016	Treatment					
	Control	62.41^c^	65.47^c^	92.50^c^	26.28^b^	18.98^c^
	LRF	83.48^b^	92.01^a^	93.57^b^	27.96^a^	30.99^a^
	LRV	104.43^a^	83.28^b^	96.65^a^	24.61^c^	22.11^b^
	Significance	**	***	***	***	***
	*t*/month					
	0	114.31^a^	73.77^c^	117.88^a^	19.44^c^	23.19^c^
	6	83.36^b^	75.26^b^	94.82^b^	27.61^b^	23.91^b^
	12	52.65^c^	91.72^a^	70.02^c^	31.81^a^	24.98^a^
	Significance	**	***	***	***	***
	T×P	**	***	***	***	***

Data were analysed using two-way ANOVA model; ns, ** and *** indicate not significant, significant at p<0.01 and p>0.0001, respectively. *t=*ageing period. T×P=significance of the interaction of treatment × period of ageing. LRF=leaf removal during flowering, LRV=leaf removal during véraison. Mean values with different letters in superscript are significantly different within treatments and period of ageing. Abbreviations: TA=total anthocyanins, HPA=total phenolic acids, TPro=total procyanidins, TFL-3-ols=total flavan-3-ols, TFG=total flavonol glycosides

**Table 4:** The effect of leaf removal on the phenolic composition of Cabernet Sauvignon wine

Year		*γ*/(mg/L)				
	TA	TPA	TPro	TFL-3-ols	TFG
2015	Treatment					
	Control	95.12^a^	47.37^b^	54.44^a^	19.23 ^b^	25.42^a^
	LRF	86.22^b^	43.73^c^	44.71^c^	21.10^a^	22.84^b^
	LRV	76.23^c^	53.42^a^	47.13^b^	19.34^b^	21.91^c^
	Significance	**	***	**	***	***
	*t*/month					
	0	138.55^a^	43.77^c^	54.09^a^	19.63^b^	32.71^a^
	6	82.86^b^	49.81^b^	48.91^b^	19.46^b^	24.92^b^
	12	36.16^c^	50.93^a^	43.28^c^	20.57^a^	12.55^c^
	Significance	**	***	**	***	***
	T×P	**	***	**	***	***
2016	Treatment					
	Control	95.75^b^	56.20^b^	39.09^c^	44.79^b^	11.30^c^
	LRF	105.90^a^	74.18^a^	61.76^a^	45.67^a^	25.96^a^
	LRV	83.95^c^	45.81^c^	40.44^b^	37.43^c^	17.88^b^
	Significance	**	**	**	**	**
	*t*/month					
	0	124.72^a^	85.36^a^	51.60^a^	17.05^c^	22.55^a^
	6	91.17^b^	33.14^c^	48.10^b^	52.09^b^	15.73^c^
	12	69.71^c^	57.68^b^	41.59^c^	58.74^a^	16.87^b^
	Significance	**	**	**	**	**
	T×P	**	**	**	**	**

Data were analysed using two-way ANOVA model; ns, ** and *** indicate not significant, significant at p<0.01 and p>0.0001, respectively. *t=*ageing period. T×P=significance of the interaction of treatment × period of ageing. LRF=leaf removal during flowering, LRV=leaf removal during véraison. Mean values with different letters in superscript are significantly different within treatments and period of ageing. Abbreviations: TA=total anthocyanins, HPA=total phenolic acids, TPro=total procyanidins, TFL-3-ols=total flavan-3-ols, TFG=total flavonol glycosides

**Table 5:** The effect of leaf removal on the phenolic composition of Syrah wine

Year		*γ*/(mg/L)				
	TA	TPA	TPro	TFL-3-ols	TFG
2015	Treatment					
	Control	105.17^c^	97.53^a^	84.97^a^	73,67^a^	69.83^b^
	LRF	115.25^b^	93.35^b^	79.41^b^	70.73^b^	82.92^a^
	LRV	134.90^a^	83.27^c^	77.34^c^	67.06^c^	68.55^c^
	Significance	**	**	**	***	**
	*t*/month					
	0	179.20^a^	84.56^c^	91.61^a^	61.07^b^	105.24^a^
	6	120.67^b^	93.45^b^	84.84^b^	58.82^c^	73.83^b^
	12	55.45^c^	96.14^a^	65.26^c^	91.55^a^	42.23^c^
	Significance	**	**	**	***	**
	T×P	**	**	**	***	**
2016	Treatment					
	Control	86.55^c^	90.71^c^	74.99^a^	27.45^c^	40.15^c^
	LRF	77.07^b^	117.40^a^	56.81^b^	29.05^b^	61.16^a^
	LRV	94.67^a^	91.19^b^	52.46^c^	31.48^a^	52.03^b^
	Significance	**	***	**	***	**
	*t*/month					
	0	128.43^a^	87.83^c^	76.72^a^	33.21^a^	61.56^a^
	6	80.25^b^	93.5^b^	59.49^b^	29.79^b^	51.77^b^
	12	49.61^c^	117.97^a^	48.05^c^	24.99^c^	43.02^c^
	Significance	**	***	**	***	**
	T×P	**	***	**	***	**

Data were analysed using two-way ANOVA model; ns, ** and *** indicate not significant, significant at p<0.01 and p>0.0001, respectively. *t=*ageing period. T×P=significance of the interaction of treatment × period of ageing. LRF=leaf removal during flowering, LRV=leaf removal during véraison. Mean values with different letters in superscript are significantly different within treatments and period of ageing. Abbreviations: TA=total anthocyanins, HPA=total phenolic acids, TPro=total procyanidins, TFL-3-ols=total flavan-3-ols, TFG=total flavonol glycosides

The positive influence of early defoliation on anthocyanin concentration due to increased UV radiation was also recorded on the Merlot in the studies by Anić *et al.* (*29*). Due to the increasingly warmer years and the influence of high temperatures on anthocyanin degradation, late leaf removal at vérasion loses its advantages over early leaf removal during flowering. Comparing the effect of both defoliation treatments, Sternard Lemut *et al*. (*39*) measured a higher concentration of total anthocyanins in Pinot Noir when the leaves were removed early, in contrast to our results for Syrah from both years.

### Anthocyanin content in wines during ageing

**Fig. 1:**
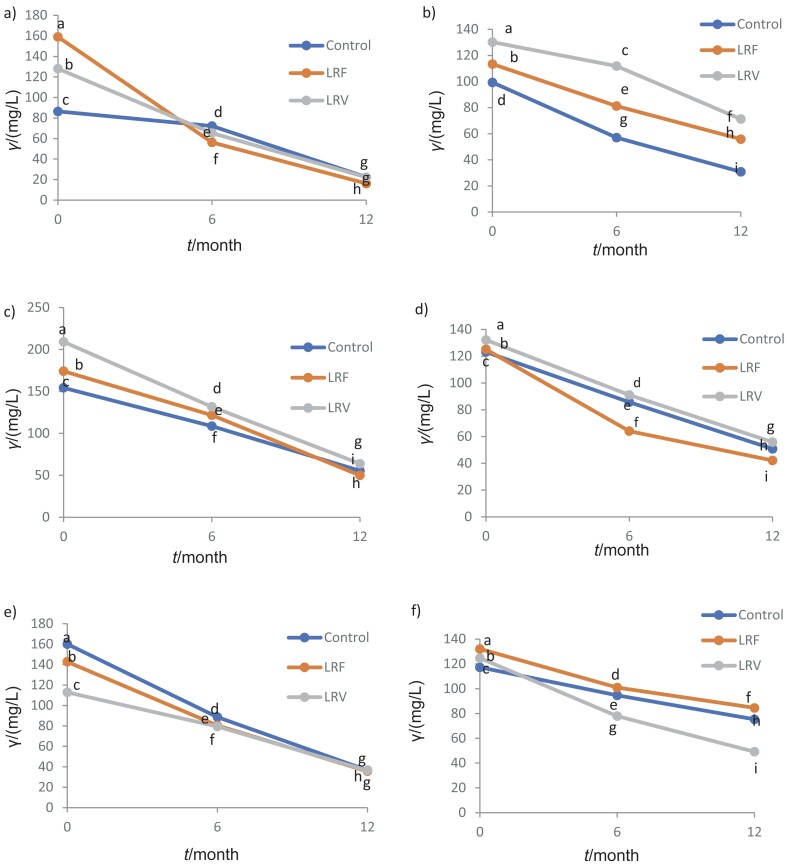
Effect of ageing and leaf removal treatment on anthocyanin content in: a) Merlot from 2015, b) Merlot from 2016, c) Syrah from 2015, d) Syrah from 2016, e) Cabernet Sauvignon from 2015 and f) Cabernet Sauvignon from 2016 wines. LRF=leaf removal during flowering, LRV=leaf removal during véraison

The wine ageing period had a significant effect on the reduction of the concentration of the individual anthocyanins in all three varieties analysed in both years (Table 6). Anthocyanin concentration decreased during wine ageing (Fig. 1), which is consistent with previous studies (*40*, *41*). Although free anthocyanins are responsible for the red colour of young red wines, their concentration decreases significantly during wine ageing to as little as 0–50 mg/L, leading to a loss of colour in red wine (*22*).

**Table 6:** Effect of ageing period shown as average values of three leaf removal treatments on the composition of anthocyanins in Merlot, Syrah and Cabernet Sauvignon wine

Wine	Year	*t*/month	*γ*/(mg/L)						
			Pet-3-Glc	Peo-3-Glc	Malv-3-Glc	Pet-3-Coum-Glc	Peo-3-Coum-Glc	Mal-3-Ac-Glc	Mal-3-Coum-Glc
Merlot	2015	0	(4.7±0.5)^a^	(1.42±0.07)^a^	(71.235.8)^a^	(1.3±0.1)^a^	(1.7±0.1)^a^	(28.4±2.4)^a^	(15.8±1.4)^a^
		6	(2.64±0.07)^b^	(0.97±0.05)^b^	(38.6±1.2)^b^	(0.57±0.02)^b^	(1.25±0.09)^b^	(1376±0.6)^b^	(6.9±0.4)^b^
		12	(0.00±0.00)^c^	(0.00±0.00)^c^	(13.2±0.7)^c^	(0.00±0.00)^c^	(1.01±0.06)^b^	(4.4±0.3)^c^	(1.6±0.1)^c^
		Significance	***	***	***	***	**	***	***
	2016	0	(3.6±0.3)^a^	(3.9±0.2)^a^	(66.5±2.2)^a^	(0.9±0.2)^a^	(1.19±0.07)^a^	(24.8±1.3)^a^	(13.4±0.4)^a^
		6	(3.2±0.3)^a,b^	(1.0±0.1)^b^	(53.6±4.8)^a^	(0.00±0.00)^b^	(0.87±0.06)^b^	(15.6±1.8)^b^	(9.0±0.9)^b^
		12	(2.4±0.3)^b^	(0.00±0.00)^c^	(35.5±3.8)^b^	(0.00±0.00)^b^	(0.00±0.00)^c^	(8.7±1.2)^c^	(6.1±0.8)^c^
		Significance	**	***	**	**	***	***	***
Syrah	2015	0	(6.0±0.2)^a^	(4.4±0.2)^a^	(94.5±4.3)^a^	(3.0±0.2)^a^	(4.9±0.2)^a^	(44.1±1.9)^a^	(22.3±4.3)^a^
		6	(4.51±0.09)^b^	(3.3±0.2)^b^	(67.4±1.9)^b^	(1.48±0.06)^b^	(2.72±0.07)^b^	(25.4±0.6)^b^	(15.9±1.7)^b^
		12	(1.18±0.04)^c^	(1.5±0.1)^c^	(31.6±1.3)^c^	(0.96±0.04)^b^	(1.2±0.1)^c^	(12.6±0.4)^c^	(6.4±1.0)^c^
		Significance	***	***	***	**	***	***	***
	2016	0	(4.0±0.2)^a^	(2.0±0.1)^a^	(69.6±1.2)^a^	n.d.	(3.3±0.3)^a^	(32.0±0.6)^a^	(17.5±0.4)^a^
		6	(2.5±0.1)^b^	(1.4±0.1)^b^	(49.5±2.4)^b^	n.d.	(1.6±0.1)^b^	(15.6±0.7)^b^	(9.6±0.6)^b^
		12	(1.61±0.09)^c^	(0.92±0.08)^c^	(31.1±1.2^c^	n.d.	(0.4±0.2)^c^	(10.0±0.3)^c^	(5.6±0.3)^c^
		Significance	***	***	***	-	***	***	***
Cabernet Sauvignon	2015	0	(3.3±0.1)^a^	(0.95±0.06)^a^	(85.5±4.4)^a^	(1.43±0.06)^a^	(1.33±0.05)^a^	(38.1±2.1)^a^	(8.0±0.5)^a^
		6	(1.97±0.03)^b^	(0.19±0.10)^b^	(52.6±1.1)^b^	(1.13±0.04)^b^	(0.84±0.04)^b^	(22.2±0.6)^b^	(4.0±0.2)^b^
		12	(0.00±0.00)^c^	(0.00±0.00)^b^	(23.4±0.1)^c^	(0.7±0.1)^c^	(0.50±0.03)^c^	(10.2±0.2)^c^	(1.45±0.09)^c^
		Significance	***	**	***	***	***	***	***
	2016	0	(1.6±0.1)^a^	(4.1±0.2)^a^	(76.4±1.5)^a^	n.d.	(1.5±0.2)^a^	(33.5±0.3)^a^	(7.5±0.3)^a^
		6	(0.03±0.03)^b^	(2.7±0.2)^b^	(58-0±2.2)^b^	n.d.	(0.96±0.06)^b^	(2498±1.2)^b^	(4.6±0.1)^b^
		12	(0.00±0.00)^b^	(2.2±0.3)^b^	(44.9±3.2)^c^	n.d.	(0.00±0.00)^c^	(18.2±1.6)^c^	(4.5±0.4)^b^
		Significance	**	**	***	-	***	***	**

Data were analysed using one-way ANOVA model and presented as mean value±S.D., *N*=9; ns,** and *** indicate not significant, significant at p<0.01 and p>0.0001, respectively. LRF=leaf removal during flowering, LRV=leaf removal during véraison. Mean values with different letters in superscript are significantly different within treatment. Abbreviations: Pet-3-Glc=petunidin-3-O-glucoside, Peo-3-Glc=peonidin-3-O-glucoside, Malv-3-Glc=malvidin-3-O-glucoside, Pet-3-Coum-Glc=petunidin-3-O-(coumaroyl) glucoside, Peo-3-Coum-Glc=peonidin-3-O-(coumaroyl) glucoside, Mal-3-Ac-Glc=malvidin-3-O-(acetyl) glucoside, Mal-3-Coum-Glc=malvidin-3-O-(coumaroyl) glucoside

The decrease in the concentration of anthocyanins in wine is partly influenced by external factors (temperature, light and precipitation). Nevertheless, some of the anthocyanins decrease due to their instability and strong reactivity with other compounds. This refers primarily to reactions of anthocyanins with other anthocyanins and their co-pigmentation and to polymerisation reactions with flavan-3-ols and procyanidins, forming new pigments of proanthocyanins and polymeric anthocyanins that can stabilise the wine colour (*19*-*21*).

According to the available literature data, previous studies confirm a steady decrease in total anthocyanin content during bottle ageing for up to 42 months (*42*-*45*). Anthocyanin degradation during ageing was high in all three wine varieties, ranging from 36 to 90 %, depending on the year and treatment. In 2016, the degradation of anthocyanins ranged from 36 to 70 %, while in 2015, the degradation was 65 to as high as 90 %, depending on the variety (Fig. 1). The highest degradation of anthocyanins was observed in Merlot wine from 2015 in the treatment of defoliation during flowering, and increased to a high 90 % after 12 months of ageing.

Anthocyanin concentration in Merlot decreased during ageing, with Pet-3-Glc, Pet-3-Coum-Glc and peonidin-3-O-coumaroyl-glucoside (Peo-3-Coum-Glc) being the most unstable. Peo-3-Glc and Pet-3-Coum-Glc were no longer detectable in any treatment after 12 months. However, the wine from the 2016 obtained after leaf removal during vérasion retained the highest total anthocyanin concentration after ageing, even though some of the individual compounds could not be detected, *i.e.* were degraded in the wines after 12 months of storage or were present in very low concentrations (Table S2).

In Syrah, the leaf removal during vérasion had the most significant positive influence on the anthocyanin content in both years (Table 5). Pet-3-Coum-Glc was undetectable in all treatments in 2016. The anthocyanin stability in the wine varied depending on the year and treatment. The treatment with the most stable anthocyanins in the wine after 12 months of ageing seems to be the 2015 control. In 2016, the control and the samples with the leaves removed during vérasion had the same effect on the stability of anthocyanins in the wine (Fig. 1 and Table S3).

The effect of ageing on the anthocyanin concentration in Cabernet Sauvignon wine is shown in Table 6. In 2015, all varieties had a significant loss of anthocyanins during ageing, while in 2016, the stability of anthocyanins in the stored wines was similar in the control and the samples defoliated during flowering. As with Syrah, Pet-3-Coum-Glc was undetectable in the samples from 2016, and Pet-3-Glc was the most unstable anthocyanin in the wine and disappeared from all wines after 12 months (Fig. 1 and Table S4).

Only in Merlot from 2016 and Cabernet Sauvignon from 2015 did the removal of leaves had a positive effect on the stability of anthocyanins. The degradation of anthocyanins in the samples defoliated during vérasion and during flowering was lower than in the control. A lower percentage of degradation was observed in the samples defoliated during vérasion.

Although there are significant differences between the anthocyanin contents in the wines after 12 months of ageing (Table 6), they cannot be related to the influence of the leaf removal treatments, regardless of their effect on the increase in anthocyanin concentration in young wines. This can be explained by the fact that the stability of anthocyanins in wine is influenced by a number of factors, such as wine storage conditions and cultivars, but also by the different reactions that anthocyanins undergo during wine ageing (*40*).

Furthermore, the degradation of anthocyanins seems to have been lower in the colder year 2016 than in the warmer year 2015. This could be explained by the differences in the basic chemical parameters, *i.e.* pH differences, since the stability and colour of red wines are strongly influenced by pH and the amount of free sulphur dioxide (*20*). The red colour of the wine comes mainly from the anthocyanins, which are in the flavylium state and whose concentration depends on the pH and the free sulphur dioxide. At a low pH, the concentration of the flavylium state increases, the hydrolysis of anthocyanins slows down and the colour is more intense, while with an increase in pH, the colour intensity and the concentration of anthocyanins in the flavylium state decrease significantly (*20*). The grapevine variety significantly influenced the total anthocyanin content in the wine. Syrah wine had the highest anthocyanin content compared to Merlot and Cabernet Sauvignon wines (Table 7).

**Table 7:** Total anthocyanin concentration in the wine after fermentation influenced by cultivar and year

Source of variation	*γ*(total anthocyanin)/(mg/L)
Cultivar	p<0.001*
Merlot	(119.4±5.7)^b^
Syrah	(153.8±7.4)^a^
Cabernet Sauvignon	(131.6±3.9)^a,b^
Year	p<0.001*
2015	(147.40±6.6)^a^
2016	(122.5±2.1)^b^

*Statistically signiﬁcant variable at p≤0.05. Results are expressed as mean value±S.E., Values with different letters in superscript are statistically different at p≤0.05

Similar results were obtained with Merlot, Syrah, Cabernet Sauvignon and Marselan in the study by Shi *et al.* (*36*). Differences in the anthocyanin content are cultivar-specific. However, the accumulation of anthocyanins in grapes is influenced by other factors, such as agroecological conditions, climate, soil conditions, canopy management and irrigation, agrotechnical practices and yield (*2*, *46*). A significantly higher concentration of total anthocyanins was found in the drier and warmer year 2015 than in 2016 (Table 7), which is in contrast to previous results (*7*, *29*), which confirm that increased solar radiation and temperature in the fruit zone reduce anthocyanin accumulation in the berry skin.

### Effects of leaf removal on other phenolic compounds in wines

Considering that anthocyanins react with other phenolic compounds in polymerisation reactions during wine ageing, other groups of phenolic compounds were analysed in all wines. In both analysed years, both leaf removal treatments increased the concentrations of total phenolic acids (TPA), total procyanidins (TPro), total flavan-3-ols (TFL-3-ols) and total flavonol glycosides (TFG) in Merlot wines compared to the control (Table 3). At the same time, such an effect was not observed in Cabernet Sauvignon and Syrah wines (Table 4 and Table 5).

This result could be a consequence of cultivar characteristics and canopy porosity, which has already been suggested by Tardaguila *et al.* (*14*). Differences were observed in the influence of the time of the leaf removal treatment, so earlier leaf removal during flowering affected the increase of TFG in Merlot and Syrah in both years, which is consistent with other studies (*29*, *32*). Defoliation increases sun exposure and UV radiation in the grape zone. Flavonols protect plants from excessive UV radiation, and their accumulation is strongly influenced by environmental conditions (*6*, *47*). Together with anthocyanins in co-pigmentation processes, flavonols form more complex compounds that affect colour stability and wine quality (*48*). Leaf removal during vérasion led to an increase in TFL-3-ol also in Merlot and Syrah wines in 2015 and 2016, which is in contrast to the results reported by Osrečak *et al*. (*33*). The end of the synthesis of flavan-3-ols in berry skin is around vérasion, so it is considered that the practice of late defoliation cannot be reflected in their concentration.

Differences in the results also exist between the two years of study, which can be related to the different meteorological and microclimatic conditions in the two vegetation seasons. Other authors also confirmed this and stated that the vintage year effect plays an important role in the successful implementation of the treatment (*37*).

### Other phenolic compounds during wine ageing

The concentration of TPA increased during wine ageing in all treatments in Syrah wines in both years (Table 5) and also in Merlot and Cabernet wines in 2015 (Table 3 and Table 4), which is consistent with previous results (*40*, *49*). The highest concentration of phenolic acids was found in the Syrah wine from 2016 and the lowest in the Cabernet Sauvignon wine from 2015. The TPro concentration in all wines decreased during ageing, except for the Cabernet control wine from 2016, and their degradation ranged from 5 to 42 %, depending on variety, year and treatment (Table S5).

The concentration of TFL-3-ols and TFG increased in some varieties, while it decreased in others during ageing. The highest concentration of TFL-3-ols was measured in Syrah wine samples defoliated during vérasion in 2015, while the highest degradation of TFL-3-ols was 33 % in Syrah samples defoliated during vérasion in 2016 and Merlot samples defoliated during flowering in 2015 (Table S5). The concentration of TFG decreased in all wines except Merlot from 2016. The lowest percentage of degradation was found in Cabernet Sauvignon wine samples defoliated during flowering in 2016, and the highest was over 70 % in Syrah samples defoliated during vérasion in 2015. The percentage of degradation of TFL-3-ols during ageing was largely influenced by the grape variety (*46*).

The interaction between the leaf removal treatment and wine ageing had a significant effect on the concentration of phenolic compounds in all three wines (Table 3, Table 4 and Table 5).

## CONCLUSIONS

The applied leaf removal treatments increased the concentration of anthocyanins in all three cultivars in both years, while the influence of leaf removal on the concentration of phenolic acids, procyanidins, flavan-3-ols and flavonol glycosides depended on cultivar and year. Leaf removal treatments had the most significant effect on the increase in the concentration of total anthocyanins in Syrah wine in both years, especially the leaf removal during véraison.

Leaf removal remains an important viticultural practice for red grapevine and wine production. Although leaf removal significantly affected the initial concentration of anthocyanins in wine, this treatment did not affect the stability of anthocyanins in the wine during ageing. Anthocyanin concentration decreases with ageing and their stability in wine was most strongly affected by ageing period and grapevine variety. Although the highest concentration of anthocyanins was found in Syrah wine, this did not affect their stability during wine ageing. Future studies should focus on how to preserve higher concentrations of anthocyanins obtained by leaf removal treatments in red wines during ageing.

## Appendix

**Table S1:** Weather conditions during vegetation period (April-September) in 2015 and 2016 (Weather station Benkovac)

Month	Average temperature/°C		Precipitation/mm	
	2015	2016	2015	2016
April	12.0	13.8	30.0	53.4
May	18.0	16.1	89.3	93.2
June	22.3	20.8	16.5	141.3
July	26.5	25.2	34.3	1.2
August	24.8	23.4	76.2	57.5
September	19.6	19.6	75.1	99.7
Mean temperature	20.5	19.8		
Cumulative precipitation			321.4	446.2

**Table S2:** Concentration of anthocyanins in Merlot wine obtained from the grapes from which leaves were removed during flowering (LRF) and during vérasion (LRV) after different ageing period (bottling, 6 and 12 months) in 2015 and 2016

Year	Treatment	month	*γ*/(mg/L)							
		*t/*	Pet-3-Glc	Peo-3-Glc	Malv-3-Glc	Pet-3-Coum-Glc	Peo-3-Coum-Glc	Mal-3-Ac-Glc	Mal-3-Coum-Glc	Total
2015	Control	0	3.1±0.1	1.18±0.02	50.14±0.04	0.77±0.04	1.19±0.04	19.3±0.1	10.75±0.07	86.4±0.4
		6	2.87±0.04	1.15±0.03	42.75±0.06	0.62±0.03	0.92±0.09	15.73±0.06	8.17±0.02	72.2±0.3
		12	n.d.	n.d.	14.74±0.09	n.d.	0.78±0.04	4.98±0.07	1.88±0.04	22.4±0.2
	LRF	0	6.27±0.03	1.66±0.05	90.55±0.07	1.73±0.05	1.96±0.07	36.04±0.09	20.77±0.05	159.0±0.2
		6	2.39±0.05	0.78±0.05	34.2±0.2	0.51±0.06	1.35±0.07	11.3±0.1	5.74±0.07	56.2±0.3
		12	n.d.	n.d.	10.5±0.2	n.d.	1.19±0.03	3.29±0.05	1.19±0.03	16.2±0.1
	LRV	0	4.67±0.2	1.4±0.2	73.0±0.2	1.3±0.2	1.8±0.2	29.8±0.2	15.9±0.2	128.0±0.2
		6	2.63±0.05	0.96±0.05	38.9±0.1	0.60±0.03	1.48±0.02	14.0±0.1	6.82±0.04	65.3±0.3
		12	n.d.	n.d.	14.40±0.04	n.d.	1.07±0.05	5.0±0.1	1.8±0.1	22.29±0.06
2016	Control	0	3.0±0.2	3.22±0.08	58.7±0.3	n.d.	1.20±0.06	21.46±0.07	11.77±0.03	99.7±0.7
		6	2.16±0.06	0.67±0.08	37.6±0.2	n.d.	0.67±0.06	10.0±0.2	6.0±0.1	57.1±0.7
		12	1.21±0.04	n.d.	21.58±0.06	n.d.	n.d.	4.9±0.1	3.1±0.1	30.8±0.3
	LRF	0	3.2±0.1	4.16±0.08	66.6±0.1	1.18±0.05	1.0±0.1	23.2±0.2	14.04±0.06	113.4±0.5
		6	3.07±0.09	0.95±0.07	52.86±0.09	n.d.	0.87±0.08	14.2±0.03	9.2±0.1	81.2±0.5
		12	2.45±0.07	n.d.	38.0±0.1	n.d.	n.d.	8.4±0.1	7.0±0.1	55.9±0.2
	LRV	0	4.76±0.07	4.33±0.05	74.1±0.1	1.53±0.08	1.41±0.06	29.9±0.2	14.25±0.04	130.2±0.4
		6	4.31±0.04	1.46±0.06	70.46±0.07	n.d.	1.09±0.06	22.54±0.06	12.0±0.1	111.8±0.3
		12	3.47±0.06	n.d.	46.85±0.06	n.d.	n.d.	12.86±0.06	8.08±0.09	71.3±0.1

Results are expressed as mean value±S.D. (*N*=3); n.d.=not detected. Abbreviations: Pet-3-Glc=petunidin-3-O-glucoside, Peo-3-Glc=peonidin-3-O-glucoside, Malv-3-Glc=malvidin-3-O-glucoside, Pet-3-Coum-Glc=petunidin-3-O-(coumaroyl) glucoside, Peo-3-Coum-Glc=peonidin-3-O-(coumaroyl) glucoside, Mal-3-Ac-Glc=malvidin-3-O-(acetyl) glucoside, Mal-3-Coum-Glc=malvidin-3-O-(coumaroyl)

**Table S3:** Concentration of anthocyanins in Syrah wine obtained from the grapes from which leaves were removed during flowering (LRF) and during vérasion (LRV) at different ageing period (bottling, 6 and 12 months) in 2015 and 2016

Year	Treatment	*t*/month	*γ*/(mg/L)								
			Pet-3-Glc	Peo-3-Glc	Malv-3-Glc	Pet-3-Coum-Glc	Peo-3-Coum-Glc	Mal-3-Ac-Glc	Mal-3-Coum-Glc		Total
2015	Control	0	5.38±0.05	4.16±0.06	79.7±0.4	2.52±0.03	4.56±0.03	38.2±0.1	19.70±0.02	154.3±0.5	
		6	4.2±0.1	3.37±0.03	60.5±0.2	1.32±0.02	2.67±0.06	23.01±0.05	13.66±0.03	108.8±0.2	
		12	1.21±0.03	1.59±0.04	29.5±0.6	0.84±0.04	1.01±0.06	12.20±0.02	6.1±0.1	52.5±0.6	
	LRF	0	5.72±0.04	3.90±0.04	94.1±0.4	3.94±0.09	4.35±0.05	42.94±0.08	19.20±0.07	174.2±0.3	
		6	4.57±0.07	2.70±0.02	68.1±0.2	1.41±0.02	2.52±0.08	25.57±0.08	16.77±0.08	121.65±0.08	
		12	1.07±0.08	1.18±0.02	28.4±0.4	0.91±0.02	1.59±0.06	11.48±0.04	5.30±0.05	50.0±0.4	
	LRV	0	6.95±0.08	5.27±0.04	109.7±0.4	2.41±0.02	5.7±0.1	51.16±0.06	27.9±0.1	209.2±0.7	
		6	4.76±0.07	3.69±0.04	73.6±0.5	1.7±0.1	2.99±0.05	27.4±0.1	17.38±0.04	131.6±0.6	
		12	1.3±0.1	1.86±0.04	36.7±0.4	1.12±0.06	1.02±0.03	14.27±0.06	7.66±0.05	63.9±0.4	
2016	Control	0	3.41±0.06	2.0±0.1	65.2±0.3	n.d.	3.42±0.06	31.76±0.08	17.26±0.04	123.2±0.3	
		6	2.63±0.07	1.64±0.06	52.4±0.5	n.d.	1.80±0.02	16.54±0.05	10.68±0.02	85.7±0.7	
		12	1.55±0.07	1.1±0.2	32.0±0.6	n.d.	n.d.	10.16±0.08	6.1±0.1	50.8±0.8	
	LRF	0	3.74±0.04	1.7±0.2	70.8±0.2	n.d.	2.35±0.09	30.04±0.06	16.48±0.03	125.1±0.4	
		6	2.08±0.08	0.95±0.09	40.0±0.4	n.d.	1.10±0.08	12.8±0.1	7.04±0.06	64.0±0.4	
		12	1.34±0.06	0.64±0.07	26.8±0.2	n.d.	n.d.	8.97±0.07	4.38±0.04	42.2±0.2	
	LRV	0	4.79±0.02	2.41±0.04	72.6±0.4	n.d.	4.20±0.05	34.15±0.06	18.85±0.04	137.0±0.4	
		6	2.91±0.04	1.66±0.06	56.0±0.1	n.d.	1.87±0.03	17.47±0.05	11.14±0.08	91.1±0.2	
		12	1.93±0.09	1.06±0.09	34.6±0.5	n.d.	1.07±0.06	10.75±0.05	6.40±0.05	55.8±0.6	

Results are expressed as mean value±S.D. (*N*=3); n.d.=not detected. Abbreviations: Pet-3-Glc=petunidin-3-O-glucoside, Peo-3-Glc=peonidin-3-O-glucoside, Malv-3-Glc=malvidin-3-O-glucoside, Pet-3-Coum-Glc=petunidin-3-O-(coumaroyl) glucoside, Peo-3-Coum-Glc=peonidin-3-O-(coumaroyl) glucoside, Mal-3-Ac-Glc=malvidin-3-O-(acetyl) glucoside, Mal-3-Coum-Glc=malvidin-3-O-(coumaroyl)

**Table S4:** Concentration of anthocyanins in Cabernet Sauvignon wine obtained from the grapes from which leaves were removed during flowering (LRF) and vérasion (LRV) at ageing period (bottling, 6 and 12 months) for 2015 and 2016

Year	Treatment	*t*/month	*γ*/(mg/L)							
			Pet-3-Glc	Peo-3-Glc	Malv-3-Glc	Pet-3-Coum-Glc	Peo-3-Coum-Glc	Mal-3-Ac-Glc	Mal-3-Coum-Glc	Total
2015	Control	0	3.57±0.04	0.82±0.04	97.9±0.3	1.29±0.05	1.46±0.05	45.1±0.2	9.84±0.07	160.0±0.2
		6	2.0±0.1	n.d.	56.9±0.1	1.02±0.07	0.76±0.07	23.9±0.2	4.2±0.2	88.7±0.2
		12	n.d.	n.d.	23.76±0.04	0.12±0.02	0.38±0.03	10.82±0.3	1.5±0.1	36.6±0.3
	LRF	0	3.5±0.2	1.2±0.1	89.8±0.5	1.32±0.03	1.40±0.02	38.5±0.3	7.06±0.09	142.7±0.8
		6	1.97±0.08	n.d.	50.5±0.2	1.14±0.01	0.97±0.08	22.68±0.08	3.2±0.1	80.6±0.3
		12	n.d.	n.d.	22.83±0.06	1.02±0.04	0.53±0.04	10.1±0.2	1.13±0.06	35.6±0.2
	LRV	0	2.77±0.04	0.87±0.04	68.76±0.04	1.67±0.04	1.1±0.1	30.7±0.7	7.0±0.1	112.9±0.7
		6	1.96±0.08	0.58±0.04	50.24±0.07	1.23±0.08	0.79±0.02	19.9±0.3	4.75±0.06	79.47±0.28
		12	n.d.	n.d.	23.58±0.09	0.89±0.03	0.59±0.04	9.57±0.08	1.74±0.03	36.4±0.1
2016	Control	0	1.14±0.08	3.25±0.09	70.93±0.1	n.d.	1.18±0.08	33.4±0.5	7.3±0.3	117.3±0.2
		6	n.d.	2.16±0.09	58.94±0.08	n.d.	0.87±0.04	27.73±0.4	4.9±0.1	94.6±0.43
		12	n.d.	1.60±0.05	47.4±0.1	n.d.	n.d.	20.9±0.2	5.36±0.09	75.3±0.3
	LRF	0	1.95±0.07	4.88±0.03	81.9±0.2	n.d.	2.0±0.1	34.7±0.3	6.64±0.04	132.2±0.5
		6	0.1±0.2	3.62±0.03	65.0±0.1	n.d.	1.20±0.03	26.7±0.4	4.3±0.2	100.9±0.6
		12	n.d.	3.46±0.06	54.4±0.3	n.d.	n.d.	21.7±0.1	5.1±0.2	84.6±0.2
	LRV	0	1.80±0.04	4.2±0.2	76.5±0.4	n.d.	1.14±0.02	32.48±0.08	8.6±0.1	124.7±0.7
		6	n.d.	2.41±0.04	50.0±0.2	n.d.	0.82±0.03	20.2±0.2	4.52±0.05	78.0±0.2
		12	n.d.	1.54±0.06	32.8±0.2	n.d.	n.d.	11.89±0.07	3.0±0.1	49.2±0.1

Results are expressed as mean value±S.D. (*N*=3); n.d.=not detected. Abbreviations: Pet-3-Glc=petunidin-3-O-glucoside, Peo-3-Glc=peonidin-3-O-glucoside, Malv-3-Glc=malvidin-3-O-glucoside, Pet-3-Coum-Glc=petunidin-3-O-(coumaroyl) glucoside, Peo-3-Coum-Glc=peonidin-3-O-(coumaroyl) glucoside, Mal-3-Ac-Glc=malvidin-3-O-(acetyl) glucoside, Mal-3-Coum-Glc=malvidin-3-O-(coumaroyl)

**Table S5:** The effect of leaf removal on the concentration of total phenolic acids, total procyanidins, total flavan-3-ols and total flavonol glycosides during different ageing period (0, 6 and 12 months) in Merlot, Syrah and Cabernet Sauvignon wines (2015/2016)

Year	Treatment	*t*/month	*γ*(total phenolic acid)/(mg/L)			*γ*(total procyanidin)/(mg/L)			*γ*(total flavan-3-ol)/(mg/L)			*γ*(total flavonol glycoside)/(mg/L)		
			Merlot	Syrah	Cabernet Sauvignon	Merlot	Syrah	Cabernet Sauvignon	Merlot	Syrah	Cabernet Sauvignon	Merlot	Syrah	Cabernet Sauvignon
2015	Control	0	52.5±0.2	83.36±0.05	43.2±0.2	93.1±0.4	92.5±0.11	59.9±0.3	22.04±0.05	56.8±0.2	21.1±0.1	37.3±0.3	91.1±0.5	35.8±0.3
		6	50.6±0.1	102.54±0.05	48.6±0.1	89.2±0.4	87.4±0.4	57.88±0.09	20.7±0.2	58.73±0.07	17.94±0.06	28.4±0.3	75.6±0.5	26.6±0.2
		12	51.4±0.1	106.7±0.1	50.3±0.1	67.3±0.3	75.0±0.4	45.5±0.2	17.3±0.2	105.5±0.5	18.6±0.5	16.3±0.4	42.8±0.1	13.8±0.2
	LRF	0	59.9±0.1	87.4±0.2	41.1±0.2	100.1±0.6	88.6±0.3	46.1±0.2	26.9±0.4	61.9±0.6	20.4±0.2	58.4±0.5	112.8±0.2	33.1±0.1
		6	60.4±0.6	95.7±0.2	44.7±0.8	77.3±0.6	83.1±0.5	43.8±0.3	22.02±0.04	59.3±0.6	20.3±0.2	32.7±0.2	83.9±0.4	23.0±0.1
		12	63.5±0.5	96.94±0.07	45.3±0.1	65.5±0.4	66.5±0.2	44.2±0.3	18.1±0.1	91.0±0.5	22.6±0.3	19.3±0.5	52.0±0.6	12.4±0.8
	LRV	0	63.3±0.4	82.95±0.1	47.0±0.4	112.7±0.3	93.64±0.03	56.2±0.3	28.84±0.08	64.6±0.4	17.3±0.1	49.4±0.2	111.7±0.7	29.2±0.4
		6	62.9±0.6	82.06±0.08	56.1±0.3	92.4±0.4	84.1±0.1	45.1±0.3	23.7±0.1	58.5±0.2	20.1±0.1	32.9±0.6	62.0±0.4	25.1±0.3
		12	62.26±0.03	84.8±0.4	57.1±0.4	81.3±0.6	54.3±0.2	40.1±0.2	20.0±0.1	78.1±0.8	20.5±0.2	18.3±0.3	31.9±0.1	11.5±0.4
2016	Control	0	60.5±0.2	79.89±0.09	81.0±0.7	110.7±0.6	98.17±0.06	34.7±0.3	17.2±0.1	30.48±0.08	14.7±0.2	15.3±0.2	41.4±0.4	13.3±0.2
		6	60.9±0.2	83.95±0.06	27.3±0.3	99.6±0.3	70.1±0.2	40.7±0.3	29.0±0.4	27.3±0.2	54.5±0.2	20.14±0.03	41.6±0.1	11.44±0.05
		12	75.0±0.3	108.3±0.3	60.3±0.3	67.2±0.3	56.7±0.5	41.8±0.1	32.6±0.2	24.6±0.2	65.2±0.1	21.5±0.1	37.4±0.5	9.2±0.2
	LRB	0	86.1±0.3	104.9±0.2	93.7±0.4	118.89±0.06	66.2±0.4	73.7±0.1	18.94±0.05	31.8±0.4	18.8±0.3	31.9±0.2	81.9±0.5	30.1±0.1
		6	84.1±0.5	110.5±0.1	38.9±0.2	92.8±0.2	58.8±0.9	60.6±0.4	28.97±0.02	30.0±0.5	55.4±0.7	30.2±0.1	60.7±0.1	20.4±0.3
		12	105.9±0.5	136.8±0.8	89.9±0.4	69.0±0.4	45.5±0.2	50.9±0.2	36.0±0.2	25.4±0.1	62.8±0.4	30.8±0.3	49.9±0.2	27.4±0.4
	LRV	0	74.8±0.4	78.7±0.6	81.45±0.09	124.0±0.1	65.8±0.2	46.4±0.8	22.1±0.2	37.3±0.4	17.7±0.3	22.3±0.8	61.3±0.2	24.2±0.3
		6	80.8±0.6	86.1±0.5	33.2±0.4	92.1±0.1	49.6±0.8	42.9±0.3	24.8±0.5	32.1±0.8	46.5±0.4	21.3±0.1	53.0±0.4	15.4±0.4
		12	94.3±0.8	108.8±0.4	22.8±0.2	73.8±0.5	41.9±0.2	32.0±0.4	26.9±0.6	25.0±0.3	48.1±0.2	22.7±0.6	41.7±0.1	14.0±0.2

Results are expressed as mean value±S.D., *N*=3
